# A practical and effective strategy in East Asia to prevent anti‐D alloimmunization in patients by C/c phenotyping of serologic RhD‐negative blood donors

**DOI:** 10.1002/jha2.292

**Published:** 2021-09-16

**Authors:** Shoichi Ito, Hitoshi Ohto, Yoshiko Ogiyama, Michiyo Irino, Susumu Omokawa, Itaru Shibasaki, Kenichi Ogasawara, Makoto Uchikawa, Kenneth E. Nollet, Willy A. Flegel

**Affiliations:** ^1^ Japanese Red Cross Tohoku Block Blood Center Sendai Japan; ^2^ Fukushima Medical University Fukushima Japan; ^3^ Japanese Red Cross Central Blood Institute Tokyo Japan; ^4^ Japanese Red Cross Kanto‐Koshinetsu Block Blood Center Tokyo Japan; ^5^ Department of Blood Transfusion and Transplantation Immunology Fukushima Medical University School of Medicine Fukushima Japan; ^6^ National Institutes of Health NIH Clinical Center Department of Transfusion Medicine Bethesda Maryland USA

**Keywords:** alloimmunization, anti‐D, DEL, red cell transfusion, RhD‐negative

## Abstract

Serologic RhD‐negative red cells can cause anti‐D alloimmunization if they carry the Asian‐type DEL or other DEL variants. *RHD* genotyping is a viable countermeasure if available, but inexpensive alternatives are worthy of consideration. RhD‐negative blood donors in Japan were studied by anti‐D adsorption‐elution and *RHD* genotyping. We collated published case reports of RhD‐negative red cell transfusions associated with inexplicable anti‐D immunization. Of 2754 serologic RhD‐negative donors, 378 were genotyped *D/d*. Anti‐D adsorption‐elution revealed 63.5% (240 of 378) to be DEL, of whom 96.7% (232 of 240) had the 1227G > A variant, diagnostic for the Asian‐type DEL. All 240 donors also carried at least one C antigen; none had a cc phenotype. The chance of transfusing DEL red cells to genuinely RhD‐negative Asian patients (based on a three‐unit transfusion) ranges from 16.7% in Korea to 69.4% in Taiwan, versus 0.6% in Germany. Among 22 RhD‐negative recipients of serologic RhD‐negative red cells, who produced new or increased anti‐D antibody titers, all 17 from East Asia were transfused with red cells with a C‐positive phenotype or known to be Asian‐type DEL or both. Serologic RhD‐negative East Asians with a cc phenotype can be red cell donors for RhD‐negative recipients, especially those of childbearing potential.

## 1 INTRODUCTION

RhD‐negative phenotypes, mostly characterized by complete deletion of the *RHD* gene, are common among Whites (15%–17%), less so among Blacks (8%), and rare in East Asians (<1%). DEL occurs among 10%–33% of East Asian individuals typed as RhD‐negative in routine serology[1]. As identified by Okubo et al in 1984, DEL has very few D antigen sites and can only be detected by specialized serology, including adsorption‐elution of anti‐D [2]. This serologic confirmation of the DEL phenotype is labor intensive.

DEL is often transfused to RhD‐negative recipients, because DEL donors are typed as RhD‐negative, as their D antigens evade detection by routine serology. Among such recipients, some experience a primary alloimmunization and develop a new anti‐D or secondary alloimmunization and increase their anti‐D titer [3, 4]. RhD‐negative patients are rare in East Asia, but cultural imperatives call for practical measures to ensure their equitable transfusion care.

Progress in genotyping enabled the molecular characterization of red cell antigens. Access to economical genotyping may become universal soon. Based on Japanese donor data, literature review (including follow‐up with authors), and biostatics, we propose in the meantime to do extended Rh phenotyping of all RhD‐negative blood donors. Blood donors who are negative for the C antigen provide a reliable source of red cells that are negative for the Asian‐type DEL and are safe for transfusion to truly D‐negative, *RHD* gene negative, recipients in East Asia.

## 2 MATERIALS AND METHODS

### 2.1 Donor samples

A total of 2754 serologic RhD‐negative blood samples donated between April and December, 2019, were investigated after initial typing with anti‐D reagent (clones TH28 and MS26, Wako, Tokyo, Japan) and automated blood grouping (PK7300 instrument, Beckman Coulter, Tokyo, Japan) at the Tohoku Block Blood Center of the Japanese Red Cross, which serves six prefectures of northeast Japan. No two samples were from the same donor.

The Ethics Committee of the Japanese Red Cross Society approved our study protocol (#2019‐038), in accordance with institutional policies, national law, and the World Medical Association Declaration of Helsinki.

### 2.2 Serology

Antiglobulin tests were done by a standard tube method with anti‐D (monoclonal LM59 IgM and 20‐175‐2 IgG: Wako), Ortho BioClone anti‐D (monoclonal IgM and polyclonal anti‐D: Ortho Clinical Diagnostics, Tokyo, Japan), and DCe/dce red blood cells (RBCs) as controls. Five monoclonal IgG anti‐D reagents (HIRO‐2, ‐3, ‐4, ‐5 and ‐55, Japanese Red Cross) were used to differentiate partial D phenotypes, including DIVb, DVa, DVI, DFR, and DYO. BioClone anti‐C, anti‐c, anti‐E, and anti‐e (all Ortho Clinical Diagnostics) were used for phenotyping C, c, E, and e, respectively.

In order to detect weakly expressed RhD antigen sites on RBCs, anti‐D adsorption‐elution was performed with two anti‐D reagents (polyclonal lot no.121104L/04‐1506: Ortho Clinical Diagnostics, and polyclonal lot no. IM‐7202: Immucor, Tokyo, Japan). Sedimented red cell samples were incubated with 10‐fold diluted anti‐D reagents for 60 min at 37°C, and after washing six times with phosphate‐buffered saline (PBS), anti‐D elution was performed with dichloromethane dichloropropan (DT‐Reagent II: Ortho Clinical Diagnostics) incubated for 5 min at 37 °C in combination with one volume of 1% bovine serum albumin‐PBS, one volume of RBCs, and two volumes of DT‐Reagent II.

### 2.3 *RHD* gene analysis

Genomic DNA was extracted from whole blood (QIAamp: Qiagen, Tokyo, Japan). The *RHD* genotype was analyzed by polymerase chain reaction (PCR), with sequence‐specific primers essentially as described previously [5]. Using PCR‐sequence‐specific primers, the *RHD* deletion (RHD*01N.01) of the hybrid *Rhesus box* and the c.1227G > A mutation of the RHD*01EL.01 were determined. Multiplex PCR was used to amplify all exons 1 through 10 of the *RHD* gene, with exon 8 as a positive control as it has no nucleotide difference between the *RHD* and *RHCE* genes. When alleles were encountered in DEL samples that could not be explained by our screening methods, *RHD* exons 1–10 were sequenced as previously described [5].

### 2.4 Likelihood of transfusing DEL red cells to genuinely RhD‐negative patients

We calculated the chance of DEL RBC transfusion as follows:

Chance of transfusing DEL red cell units = 1‐[X]^n^,

where X is the fraction of truly RhD‐negative (lacking the *RHD* gene) among serologic RhD‐negative red cell units, and N is the number of red cell units transfused from different blood donors.

### 2.5 Literature review of cases with anti‐D alloimmunization

We searched for published case reports of transfusion‐related anti‐D formation, reported in Japanese or English languages, using Japana Centra Revuo Medicina, and PubMed, as well as meeting abstracts of the Japan Society of Blood Transfusion and Cell Therapy (formerly the Japan Society of Blood Transfusion) and the AABB (formerly the American Association of Blood Banks). Additional information was solicited from authors via email when available. Data abstracted from relevant cases included 1. patient anti‐D titers, 2. history of blood transfusion or pregnancy, and 3. DEL‐associated information about transfused red cell units.

## 3 RESULTS

### 3.1 *RHD* genotype, *RHD* transcripts, and RhD phenotype

Among 2754 samples apparently RhD‐negative by routine serology, 378 (13.7%) genotyped as *D/d*, indicative of being RhD‐positive, whereas the remaining 2376 (86.3%) were genotyped *d/d*, or truly RhD‐negative (Table 1‐A). Anti‐D adsorption‐elution of the 378 with genotype *D/d* identified 240 (63.5%) as DEL (Table 1‐B), leaving 138 (36.5%) for which further studies were not performed.

**TABLE 1‐A:** *D/d* genotypes among 2754 serologically RhD‐negative blood donors

Genotype	*d/d*	*D/d*
Phenotypically D‐negative donor samples	2,376 (86.3%)	378 (13.7%)

**TABLE 1‐B:** Anti‐D adsorption‐elution results on 378 blood samples of *D/d* genotype

Adsorption‐elution results	Test positive	Test negative
Donor samples with *D/d* genotype	240 (63.5%)	138 (36.5%)

**FIGURE 1:**
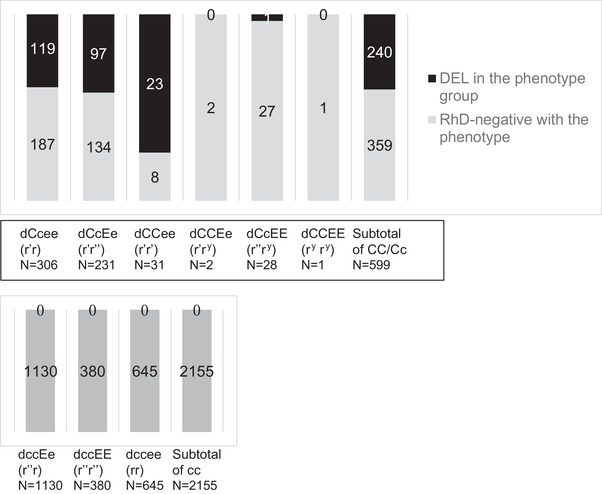
Serologic characteristics of 2754 blood samples that typed RhD‐negative in routine serology. The DEL phenotype is detected among C antigen positive samples (upper panel). No DEL phenotype was observed among C antigen negative samples (lower panel)

Of 2754 phenotypically RhD‐negative donors, 597 (21.7%) carried the C antigen whereas the remaining 2155 (78.2%) did not (Figure 1). Further, 240 (40.2%) of the 597 with C antigen were DEL, while none of the 2155 without C antigen had genotyped as *D/d*.

### 3.2 Relationship between DEL and the C/c phenotypes

Of the 240 DEL in our cohort, 232 (97%) had the 1227G > A variant, diagnostic for the Asian‐type DEL, whereas eight had other *RHD* alleles (Table 2). Among these eight, all of which were RhC‐antigen positive, one was found to have a new allele: c.1228‐1G > A at intron 9.

**TABLE 2:** Serological characteristics and nucleotide mutations in eight of 240 donor specimens that did not harbor the 1227G > A variant, diagnostic for the Asian‐type DEL

Sample	Rh phenotype	Anti‐D adsorption‐ elution	Nucleotide change	Amino acid change	Exon/intron
D‐0271	CcEe (r'r’’)	1+	c.1213C > T	p.Gln405Stop	Exon 9
D‐2139	Ccee (r'r)	2+^s^	c.1252T > A	p.Stop418Lysext26	Exon 10
D‐0660	Ccee (r'r)	2+^s^	c.721A > C	p.Thr241Pro	Exon 5
D‐1256	CcEe (r'r’’)	2+	Under investigation		
D‐1584	Ccee (r'r)	2+	Under investigation		
D‐1486	CcEe (r'r’’)	2+^s^	c.1252T > A	p.Stop418Lysext26	Exon 10
D‐1676	CcEE (r’’r^y^)	2+^s^	c.1228‐1G > A (new)	Splicing failure	Intron 9
RH‐280	CcEe(r'r’’)	2+^s^	c.1222T > C	p.Trp408Arg	Exon 9

### 3.3 Likelihood calculation for transfusing DEL red cells to genuinely RhD‐negative patients

Genuinely RhD‐negative patients, who do not harbor an *RHD* gene, occur in East Asia with frequencies ranging between 0.19% among Chinese in Hong Kong, 0.23% of both Koreans and Chinese in Taiwan, and 0.46% of Japanese [6– 9]. Based on these data, the chance of transfusing DEL red cells to genuinely RhD‐negative recipients, assuming a 3‐unit exposure, ranges between 16.7% in Korea and 69.4% in Taiwan (Table 3). This likelihood was substantially higher than the 0.6% calculated for Germany, representative of Caucasian populations [10].

**TABLE 3:** The prevalence of RhD‐negative and DEL phenotypes in East Asia and Europe

					Chance of transfusing DEL red cells to genuinely D‐negative patients receiving phenotypically D‐negative red cells by routine serology		
Country/region	Phenotypically D‐negative prevalence in the general population (/10,000) [A]	Prevalence of DEL among those phenotypically D‐negative in the general population [B]	Reference	Genuinely D‐negative prevalence in the general population[C] = [A]x(1‐[B]/100)	One unit	Three units	Five units
Japan	0.50% (50)	8.7%	This study and [6]	0.46%	8.7%	23.9%	36.6%
China (Hong Kong)	0.27% (27)	29.3%	[7]	0.19%	29.3%	64.7%	82.3%
Taiwan	0.34% (34)	32.6%	[8]	0.23%	32.6%	69.4%	86.1%
Korea	0.24% (24)	5.9%	[9]	0.23%	5.9%	16.7%	26.2%
Germany	17.0% (1700)	0.21%	[10]	17.0%	0.21%	0.6%	1.1%

### 3.4 Literature review of case reports

Reports from 2005 to 2021 include 22 RhD‐negative transfusion recipients of serologic RhD‐negative RBCs producing a new anti‐D or an increase in anti‐D titer with or without having a hemolytic transfusion reaction (Table 4). Among them, nine were Japanese, and six were Chinese patients (including in Taiwan); two patients were reported in Korea, including a Russian patient. The remaining five included four in Canada, and one in Austria. More female (*n* = 14) than male patients (*n* = 8) were documented.

**TABLE 4:** Reports of primary and secondary anti‐D sensitization by red cell transfusion

Case	Country/region	Age (years), sex (F/M)	Previous transfusion/pregnancy	Primary/secondary response	Transfusion‐associated hemolysis	Transfused RBCs (DEL or DEL suspected)	Reference year
1	Japan	67 F	Transfusion in her 20s	Secondary (Neg→ titer 8 →128)	No hemolysis	2 bags DEL 1227G > A	[4] 2005
2	Japan	70s F	Pregnancy (3)	Secondary (Neg→ pos, 34 days)	No hemolysis	1 bag DEL 1227G > A	[11] 2006
3	Japan	57 F	Pregnancy (1, D+)	Secondary (Neg→ 64, 2 years→ 4096, +5 months)	No hemolysis	2 bags DEL (Ccee, CcEe), both 1227G > A	[12] 2010
4	Japan	70s F	Pregnancy	Secondary (Neg→ pos, 28 days) (anti‐D+ anti‐C)	Not reported	1 bag C (+)	[13] 2011
5	Japan	85 F	Pregnancy	Secondary (Neg→ titer 1, day 4→ 8, day 11)	Not reported	1 bag Ccee	[14] 2012
6	Japan	35 M	None	Primary (Neg→ pos, 5 months, anti‐D+ anti‐C)	Not reported	1 bag DEL	[15] 2015
7	Japan	79 M	Transfusion in his 20s	Secondary (Neg→ titer 1, day 4 → 8, day11)	No hemolysis	1 bag DEL 1227G > A	[16] 2015
8	Japan	86 F	Pregnancy (4, D+)	Secondary (Neg→ pos, day 9)	No hemolysis	1 bag Ccee	[17] 2019
9	Japan	80s F	Pregnancy and transfusion	Secondary (Neg→2, 2 weeks)	No hemolysis	DEL 1227G > A	[18] 2021
10	China	33 F	Pregnancy (2)	Secondary (titer 8 →64)	Delayed hemolytic transfusion reaction	DEL	[19] 2012
11	China	45 M	Transfusion, 20 years ago	Secondary (titer 8 →64)	No hemolysis	DEL	[19] 2012
12	China	28 F	Pregnancy (D+, 3) and transfusion (D+)	No change in titer (512→512)	Delayed hemolytic transfusion reaction	2 units DEL	[19] 2012
13	China	68 M	None	Primary (Neg→ titer 2, day 22)	Not reported	DEL	[19] 2012
14	Taiwan	64 M	No information available	Primary? (Neg→ pos, 1 month)	Not reported	DEL (2 bags of C positive)	[20] 2006
15	Taiwan	73 M	No information available	Secondary? (Neg→ pos, 6 days)	Not reported	DEL (4 bags of C positive)	[20] 2006
16	Korea	68 M	None	Primary (Neg→ pos, 9 days)	No hemolysis	1 bag DEL 1227G > A	[21] 2009
17	Korea (Russian)	64 M	None	Primary (Neg→ pos, 7 days)	No hemolysis	2 bags (Ccee, CCEe) DEL 1227G > A	[22] 2015
18	Austria	58 F	Pregnancy (1)	Primary (Neg→ pos, 8 days →64‐128, 1 year)	No hemolysis	DEL (*RHD* IVS5‐38del4)	[3] 2005
19	Canada	88 F	Pregnancy	Secondary (Neg→ pos, 4 weeks)	No hemolysis	DEL (RHD*(93‐94insT))	[23] 2013
20	Canada	87 F	Pregnancy and transfusion (1 RhD+ platelets)	Secondary (Neg→ pos, several weeks)	No hemolysis	DEL (RHD*(93‐94insT))	[23] 2013
21	Canada	88 F	Pregnancy and transfusion (1 RhD+ platelets)	Secondary (Neg→ pos, several weeks)	No hemolysis	DEL (RHD*(93‐94insT))	[23] 2013
22	Canada	44 F	Transfusion (1 RhD+ platelets and 6 whole blood platelets)	Secondary (Neg→ pos, several weeks)	No hemolysis	DEL (RHD*(93‐94insT))	[23] 2013

Of 17 case reports in East Asia, all 17 were apparently transfused with at least one red cell unit of Asian‐type DEL or RhC‐positive phenotype or both. No recipient in Europe or Canada received Asian‐type DEL red cell units, but did receive other DEL variants (Table 4).

Among 17 East‐Asia type DEL red cell transfusion recipients with primary or secondary alloimmunization, only two developed post‐transfusion delayed hemolytic transfusion reactions, whereas the remaining 15 did not experience any hemolysis or other transfusion reaction (Table 4). Among these 17 recipients, five were presumed to have experienced a primary, and 12 a secondary alloimmunization (Table 4).

## 4 DISCUSSION

Individuals with an RhD‐negative phenotype are rare in East Asia, accounting for 0.50% of Japanese [6], 0.27% of Chinese in Hong Kong [7], 0.34% of Taiwanese [8], and 0.24% of Koreans [9], versus 15%–17% of Whites and 8% of Blacks [6, 10]. Among serologic RhD‐negative Japanese, adsorption‐elution testing documents 8.7% carrying a DEL; hence 91.3% of serologic RhD‐negative Japanese, or 0.46% of the general population in Japan, are genuinely RhD‐negative (lacking the *RHD* gene).

All 240 DEL samples in our cohort of 2754 serologic RhD‐negative blood donors carried the CC or Cc phenotypes, without any samples carrying the cc phenotype. Hence, 40% (240 of 599) of CC/Cc samples were DEL, but none of 2155 cc samples were DEL. This observation from a large Japanese donor cohort is consistent with a Chinese study from Hong Kong, where all of 136 DEL donors carried either the CC or Cc phenotypes, but none carried the cc phenotype [7]. A similar association had been observed in non‐Asian populations [24, 25].

**FIGURE 2:**
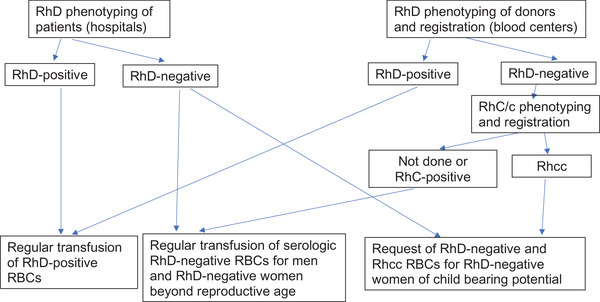
Proposed work‐flow for testing routine serologic Rh testing. The RhD phenotyping for patients (left side) follows current practice in Japan, but implements a special request of C‐negative blood units for persons of childbearing potential. The RhD phenotyping for donors (right side) requires additional serologic testing for the C and c antigens. The results will be registered in the donor database and used to fill requests for C‐negative red cell units that are expected to be DEL negative (see Figure 1)

We propose to perform extended Rh phenotyping on the few blood donors who are serologic RhD‐negative on initial routine testing, and then exclusively supply red cell units with a cc phenotype to RhD‐negative patients when indicated, as shown in Figure 2. As approximately 80% of serologic RhD‐negative East Asian blood donors (78% of Japanese and 82% of Chinese [7]) also carry the cc phenotype, this practical and effective strategy would not significantly alter the supply and demand calculus of RhD‐negative red cells for RhD‐negative patients in East Asia. It will increase patient safely and reliably prevent anti‐D alloimmunizations by blood transfusion.

In Japan, we have identified only eight patients with primary or secondary transfusion‐associated anti‐D alloimmunizations in the 15 years since 2006 hitherto unpublished surveillance), an average of less than one such patient per year. Among 1,000,000 annual transfusion recipients, a 0.46% prevalence of genuinely RhD‐negative would amount to 4600 patients, of whom approximately 1000 (24%) would be exposed to DEL red cells, assuming 3‐unit red cell transfusions. Of those, 700 (70%) would likely be alive at 6 months and beyond. With more than a half of them (350) having post‐transfusion antibody screening, the reported alloimmunization incidence is less than 1 (0.3%). Even with under‐recognition and under‐reporting, we recognize that the immunogenicity of DEL is quite low compared to regular RhD‐positive red cells. The RhD antigen is the most immunogenic of all Rh and other non‐ABO red cell antigens. The demonstrated low immunogenicity of DEL can be explained, at least in part by the extremely low number of <30 D‐antigen sites per DEL red cell [26], compared to approximately 10,000–>33,000 per normal D‐positive [27] and 300 – 5000 for weak D red cells [28].

Tsutsui and colleagues identified 26 women with anti‐D among 1811 RhD‐negative female donors in Japan (1.4%), who were 16–65 years of age without a history of transfusion [29]. Almost all subsequent pregnancies would involve RhD‐positive babies at risk of hemolytic disease of the fetus and newborn. The possible clinical impact is unknown, as no systematic follow‐up and reporting is mandated in Japan. Rh immunoglobulin (RhIG) administration was introduced in the 1970s and is now used nationally in Japan's system of universal healthcare to prevent of RhD sensitization of RhD‐negative women with RhD‐positive pregnancies. Failed RhD prophylaxis may be attributed to massive feto‐maternal transfusion and non‐administration of RhIG in the context of spontaneous or elective abortion. RhIG is not indicated for mothers delivering RhD‐negative neonates, but a DEL fetus, more common than routinely detected, could cause an anti‐D sensitizing event. As a precautionary countermeasure, currently available C/c phenotyping, a standard practice in many European countries, would easily and inexpensively mitigate potentially adverse outcomes.

Our aggregated data of Asian‐type DEL red cell transfusions (Table 4) document only two of 17 recipients who developed post‐transfusion hemolytic transfusion reactions. Based on this clinical data, we propose to prioritize red cell units of cc phenotype, because they are scarce, for transfusion to recipients of childbearing potential, rather than to any RhD‐negative patient.

In conclusion, while the technology and economy of genotyping progresses, it is practical, economical, and effective in East Asia to implement C/c phenotyping of all serologic RhD‐negative blood donors, and select those who are C‐negative (cc phenotype) for red cell transfusion to RhD‐negative patients, predominantly those of childbearing potential. This approach may be useful in other population as well, either for routine care or in the context of a crisis in which genotyping is unavailable.

## AUTHOR CONTRIBUTIONS

All have contributed sufficiently to be included as authors. Shoichi Ito, Yoshiko Ogiyama, Michiyo Irino, Kenichi Ogasawara, and Makoto Uchikawa performed research, data analysis, and data interpretation. Hitoshi Ohto collected the clinical case reports. Susumu Omokawa, Itaru Shibasaki, and Willy A. Flegel supervised the study. Hitoshi Ohto, Kenneth E. Nollet, and Willy A. Flegel collaborated in the writing and critical revision of the manuscript.

## CONFLICT OF INTEREST

The authors declare no conflicts of interest.
